# Cancer-related post-treatment pain and its impact on health-related quality of life in breast cancer patients: a cross sectional study in Palestine

**DOI:** 10.1186/s12930-017-0037-3

**Published:** 2017-11-21

**Authors:** Nader H. Abu Farha, Mohammed T. Khatib, Husam Salameh, Sa’ed H. Zyoud

**Affiliations:** 1 Department of Medicine, College of Medicine and Health Sciences, An-Najah National University, Nablus, 44839 Palestine; 2 Poison Control and Drug Information Center (PCDIC), College of Medicine and Health Sciences, An-Najah National University, Nablus, 44839 Palestine; 3 Division of Clinical and Community Pharmacy, Department of Pharmacy, College of Medicine and Health Sciences, An-Najah National University, Nablus, 44839 Palestine

**Keywords:** Breast cancer, Post-treatment pain, Health-related quality of life, Palestine

## Abstract

**Background:**

Post-treatment pain has been suggested as an important indicator for health-related quality of life (HRQOL) in patients with breast cancer. Therefore, this study was performed to examine the association between pain and its impact on HRQOL among breast cancer patients in Palestine. Also, this study aimed to determine the QOL profile for breast cancer patients and stated the factors associated with QOL.

**Methods:**

A correlational cross-sectional study was conducted from May 2016 to November 2016 at Al-Watani Hospital and An-Najah National University Hospital in the Nablus district in Palestine. The five-level EuroQol five-dimensional instrument (EQ-5D-5L) was used to examine HRQOL. Pain severity and interference were assessed using the Brief Pain Inventory (BPI). Multiple linear regression analysis was performed to determine the most important variables related with HRQOL.

**Results:**

One hundred and seventy patients were involved in this study. Overall, all participants were female, with a mean ± SD for age of 51.71 ± 11.11 years. The reported HRQOL of this study was measured by using the median EQ-5D-5L index score, which was 0.67 (interquartile range: 0.51–0.84). There were moderate negative correlations between EQ-5D-5L index score and pain severity score (r = − 0.58, *p* value < 0.001), and pain interference score (r = − 0.604, p-value < 0.001). Furthermore, univariate analysis showed that age, marital status, employment status, income, current condition of cancer, and post-treatment pain were associated with quality of life (p-value < 0.05). Regression analysis revealed that patients with high income (p-value = 0.003), patients with lower pain severity score (p-value < 0.001), and lower pain interference score (p-value = 0.018) were independently associated with high QOL.

**Conclusions:**

This is the first study to present important data regarding QOL by using the EQ-5D-5L instruments that may help healthcare providers to identify patients at risk of low QOL. Healthcare providers and health strategy makers should be alerted to low level HRQOL among breast cancer patients with low income level, patients with post-treatment pain, especially in the state of severe pain, and the state of pain interfering with daily life to improve their HRQOL.

## Background

Breast cancer refers to a malignancy in women and in a small percent in men, which arises from the epithelial tissue of the breast tissue, representing approximately 10% of the total volume of the breast [1]. Breast cancer is the second most common cancer globally, and also the most common malignancy between women that consists of 18% of all female cancers [1, 2]. Breast cancer has different treatment methods, and these methods have different effects on the patients and their life [3]. The treatment of breast cancer usually starts with surgery and radiotherapy, and often involves chemotherapy or other drug therapies, such as hormonal treatment, either before or after surgery [4]. Pain after treatment is a major clinical problem in breast cancer patients, and is one of the most common complications affecting 25 to 60% of breast cancer patient survivors [5]. Post-treatment pain is defined as the pain related to treatment body regions with duration of more than 3 months after treatment is completed [6]. Improving health-related quality of life (HRQOL) has become one of the most essential goals of cancer therapy [7–9]. HRQOL is a multidimensional instrument that includes the comprehension of the positive and the negative aspects of different dimensions such as the physical, emotional, cognitive and social domains, as well as pain/discomfort [10].

In Palestine, cancer is the second most common cause of death, accounting for about 14.2% of all deaths in 2014, meaning that they are very common [11]. According to the Ministry of Health records, breast cancer is the most common type of cancer in Palestine and the third most common type of cancer causing death (about 10.7%) after lung and colon cancer [11]. Globally, there are many articles that talk about post-treatment pain and HRQOL among breast cancer survivors [5, 6, 12–17]. In the Arab World and Palestine, there is no research related to the post-treatment pain and its association with HRQOL in breast cancer patients. Researches in Palestine about breast cancer focused on the palliative care situation [18] and pharmacological treatment [19, 20]. Therefore, this study was performed to examine cancer-related post-treatment pain (pain severity and interference) and its impact on HRQOL in the different stages of breast cancer in patients in Palestine. Also, this study aimed to determine QOL profile among breast cancer patients and stated the factors associated with QOL. Investigating and assessing QOL in breast cancer patients and the related post-treatment pain will help medical teams and patients to plan and develop spectacular pain management strategies to address common signs and symptoms, and provide breast cancer patients with better health and good QOL. Also, this will assist in creating a complete system in order to deal with current patients and future patients, so that we can help to end the suffering of these patients.

## Methods

### Study design

This cross-sectional study was conducted by using standardised and validated assessment tools in women with breast cancer from May 2016 to November 2016.

### Study setting

This study was conducted in Al-Watani hospital and An-Najah National University hospital, Nablus, West Bank, Northern Palestine. These two hospitals serve as the main referral hospitals for the northern districts of West Bank-Palestine and receive most cases of breast cancer patients from all northern West Bank districts.

### Study population

The medical records of both hospitals in 2015 showed that the number of breast cancer patients in both hospitals was around 600 patients in 1 year and around 300 patients during the period of study. Each of both hospitals where study was conducted gave us a list with the names of breast cancer patients in order to assess their comfort for this study.

### Sampling procedure and sample size calculation

The Raosoft sample size calculating tool (an automated software program: http://www.raosoft.com/samplesize.html) was used for sample size calculation. We assumed that 50% of women with breast cancer had a high QOL, which would give the maximum sample size. Furthermore, we used a 5% margin of error at a 95% confidence interval as recommended; the required sample size was calculated to be 170 women. Convenience sampling was used to recruit participants.

### Inclusion and exclusion criteria

Women aged 18 years and above who were treated for breast cancer > 12 months prior to the conduct of our study, and who agreed to be participants in this study were included. The only exclusion criteria were women who had a major psychiatric illness, and those with an extremely ill condition.

### Data collection instrument

The data collection form consisted of four sections:

1. The first section was designed to obtain socio-demographic data such as age, marital status, place of residence, educational level, family monthly income, and height and weight, to calculate body mass index (BMI).
2. The second section contained patient clinical data such as type of breast cancer, stage of breast cancer, duration of disease, and the types of management that the patient had undertaken.
3. The third section was based on the assessment of post-treatment pain and discomfort among breast cancer patients by using a well-known pain-measuring scale which is called the Brief Pain Inventory scale (BPI); [21]. The BPI was used to assess both pain severity and pain interference with normal functioning. Items used in determination of pain severity were worst pain in the last 24 h, least pain in the last 24 h, average pain in the last 24 h and pain right now. Seven items were designed to assess pain interference with general activity, walking ability, mood, normal work, sleep, relations with others, and enjoyment of life. Also, this scale determined pain location (head, right breast, left breast, abdomen, right upper limb, left upper limb, back, knees, ankle and feet and buttocks), pain relief by medication and percentage of pain relief. Pain severity score was measured by the sum of 4 items of pain severity. Each item was scored as a number from 0 to 10, and the sum of these numbers gave the final pain severity score with the lowest value of 0 and the highest value of 40. In addition, pain interference score was measured by the sum of the 7 items of pain interference. Each item was scored as a number from 0 to 10, and the sum of these numbers gave the final pain interference score with the lowest value of 0 and the highest value of 70. Permission was obtained from the Department of Symptom Research at the University of Texas to use the Arabic Brief Pain Inventory in our study.
4. The fourth section consisted of the EQ-5D instrument to assess HRQOL. EQ-5D is a widely used instrument for evaluation of the generic quality of life [22]. EQ-5D is a preference-based HRQOL measure; it includes one question for five dimensions: mobility, self-care, normal activities, pain/discomfort, and anxiety/depression [23]. Moreover, the EQ-5D questionnaire also has a Visual Analog Scale (VAS); by using this scale, respondents can report and document their perceived health status by a grading system ranged from 0 (the worst possible health status) to 100 (the best possible health status). The Arabic version of EQ-5D [24] was provided by the EuroQol Research Foundation [23] through registration on the EQ-5D online system (ID: 15804). This scale has been described in detail in many previous studies conducted by the principle investigator [25–27]. The EQ-5D index scores were calculated as illustrated elsewhere [27–31], using the EQ-5D-5L Crosswalk Index Value Calculator [32] based on the UK general population scoring algorithm.

Academic experts (two clinical pharmacists with expertise in QOL research and one academic researcher with experience in statistical analysis) reviewed and evaluated measurement items for face and content validity, and clinical accuracy. Data collection forms were administered to participants face-to-face by two medical students. These researchers received training in investigation skills and research ethics at the College of Medicine and Health Sciences and from epidemiologists with expertise in quality of life research. In order to insure interviewer consistency, both of the interviewers interviewed the participants closely with each other. The data collection form was piloted on 15 patients (not included in the final study) to assess questionnaire comprehension, clarity, and completion time. The results of the pilot study were evaluated critically and some minor modifications were made accordingly for socio-demographic and clinical data.

### Ethical approval

The Institutional Review Board (IRB) of An-Najah National University (#20Mar2016) approved the study. Permission was obtained from the two selected hospitals for allowing researchers to interview their patients.

### Statistical analysis

Analysis of data was done with the IBM Statistical Package for Social Sciences (SPSS, version). Continuous variables were presented mainly as mean ± SD or medians (lower–upper quartiles), and categorical variables were both expressed as frequency and percentage. Normality of continuous data was checked by the Kolmogorov–Smirnov test. Continuous variables such as the EQ-5D-5L index score was tested for intra-individual differences by using the Kruskal–Wallis or Mann–Whitney test, as required. In addition, the Spearman correlation coefficient was used to assess the degree of association between all scales. The significance level was determined at a p-value < 0.05. Multiple linear regression analysis was also used to determine independent associations with HRQOL. Variables (socio-demographic, clinical, and pain severity and interference) that were significant in bivariate analysis were entered into regression models. Cronbach’s alpha was assessed for each scale to check the scale’s internal consistency reliability.

## Results

### Socio-demographic and clinical characteristics

One hundred and eighty-three patients were interviewed, and the response rate was 92.9%. In total, 170 patients (all females; mean age 51.71 ± 11.11 years) with breast cancer were recruited for the study. Of these, 67 (39.4%) patients were aged between 50 and 59 years old. Ninety-one (53.5%) participants lived in villages and 132 (77.6%) were married. More than 80% of the participants were housewives, and 75 (44.1%) participants lived in families with a moderate income level. The socio-demographic data of the study participants are listed in Table 1.

**Table 1:** Socio-demographic status and health-related quality of life

Variable	n (%) N = 170	Median EQ-5D-5L index (1st percentile-3rd percentile)	*P* value
Age (year)			
< 40	21 (12.4)	0.71 [0.54–0.77]	*0.043* ^a^
40–49	48 (28.2)	0.76 [0.55–0.88]	
50–59	67 (39.4)	0.67 [0.45–0.84]	
> 60	34 (20.0)	0.58 [0.46–0.69]	
Residency			
City	64 (37.6)	0.66 [0.52–0.83]	0.966^a^
Village	91 (53.5)	0.68 [0.49–0.84]	
Palestinian refugee’s campaign	15 (8.8)	0.63 [0.45–1.00]	
Marital status			
Single	38 (22.4)	0.56 [0.41–0.67]	*<* *0.001* ^b^
Married	132 (77.6)	0.71 [0.54–0.88]	
Educational level			
Elementary	20 (11.8)	0.52 [0.41–0.77]	0.363^a^
Preparatory	55 (32.4)	0.68 [0.58–0.85]	
Secondary	53 (31.2)	0.71 [0.54–0.88]	
Diploma	20 (11.8)	0.66 [0.32–1.00]	
Bachelor’s degree	10 (5.9)	0.66 [0.42–0.72]	
Uneducated	12 (7.1)	0.68 [0.47–0.70]	
Occupational status			
Private employee	16 (9.4)	0.74 [0.49–1.00]	0.294^a^
Government employee	13 (7.6)	0.62 [0.32–0.72]	
Housewife	141 (82.9)	0.68 [0.52–0.84]	
Income level			
Low (less than 500 JD)	65 (38.2)	0.58 [0.41–0.74]	*0.002* ^a^
Moderate (500 JD–1000 JD)	75 (44.1)	0.70 [0.58–0.88]	
High (more than 1000 JD)	30 (17.6)	0.76 [0.53–0.88]	
Body mass index			
Underweight (< 18.5)	6 (3.5)	0.41 [(− 0.43)–0.68]	0.065^a^
Normal weight (18.5–24.9)	48 (28.2)	0.60 [0.46–0.84]	
Overweight (25–29.9)	70 (41.2)	0.71 [0.58–0.84]	
Obese (> 30)	46 (27.1)	0.69 [0.44–0.85]	

Italic values indicate significance of *p* value (*p* < 0.05)

^a^Statistical significance of differences calculated using the Kruskal–Wallis test

^b^Statistical significance of differences calculated using the Mann–Whitney U test

As shown in Table 2, the majority of patients had the invasive ductal carcinoma (IDC) histopathological type breast cancer; with 159 (93.5%) patients having IDC compared with other types of breast cancer, like ductal carcinoma in situ (DCIS) and lobular ductal carcinoma (LDC). With regard to breast cancer treatment, 165 (97.1%) patients were taking one or more chemotherapy agents, 139 (81.8%) patients had undergone breast surgery, 62 (36.5%) patients had received radiotherapy and 59 (34.7%) patients had received hormonal therapy. The most commonly used chemotherapy protocols were Cyclophosphamide + Adriamycin and Taxol which were used in 96 (56.5%) and 80 (67.1%) patients, respectively. “Total mastectomy with some or total removal of the axillary lymph nodes surgery” was the most common type of surgery, used in 105 (61.8%) patients. The current breast cancer condition shows that 60 (35.3%) patients are cancer-free, 58 (34.1%) and 47 (27.6%) patients are at stage 1 and stage 4, respectively, and 104 (61.2%) had received treatment in the last 3 months before participation in the study.

**Table 2:** Cancer current condition and health-related quality of life

Variable	n (%) N = 170	Median EQ-5D-5L index (1st percentile-3rd percentile)	*P* value
Type of breast cancer			
Invasive ductal carcinoma	159 (93.5)	0.67 [0.52–0.84]	0.419^a^
Invasive lobular carcinoma	6 (3.5)	0.49 [0.25–0.77]	
Ductal carcinoma in situ	5 (2.9)	1.00 [0.81–1.00]	
Stage of cancer^c^			
Stage 1	58 (34.1)	0.72 [0.55–0.85]	0.125^a^
Stage 2	24 (14.1)	0.61 [0.52–0.77]	
Stage 3	40 (23.5)	0.67 [0.51–0.81]	
Stage 4	47 (27.6)	0.60 [0.37–0.88]	
Current condition			
Cancer-free	60 (35.3)	0.74 [0.55–0.85]	*0.023* ^a^
The tumor returned	30 (17.6)	0.62 [0.51–0.85]	
Active and receiving treatment	80 (47.1)	0.64 [0.37–0.77]	
Last time received treatment			
0–3 months	104 (61.2)	0.65 [0.41–0.84]	0.310^a^
3–12 months	16 (9.4)	0.59 [0.51–0.77]	
1–2 years	24 (14.1)	0.74 [0.63–0.84]	
More than 2 years	26 (15.3)	0.67 [0.53–0.86]	
Post-treatment pain			
Yes	149 (87.6)	0.65 [0.48–0.80]	*0.013* ^b^
No	21 (12.4)	0.85 [0.58–1.00]	

Italic values indicate significance of *p* value (*p* < 0.05)

^a^Statistical significance of differences calculated using the Kruskal–Wallis test

^b^Statistical significance of differences calculated using the Mann–Whitney U test

^c^Unknown stage of cancer for one case

### Brief Pain Invitatory

The median pain severity score was 14.50 (interquartile range: 8.00–21.25), and the median pain interference score was 17.00 (interquartile range: 9.00–30.00). Reliability values for these two subscales were good (Cronbach’s alpha = 0.895 and 0.879, respectively).

### EQ-5D health status, EQ-5D-5L index score, and EQ-VAS score

**Fig. 1:**
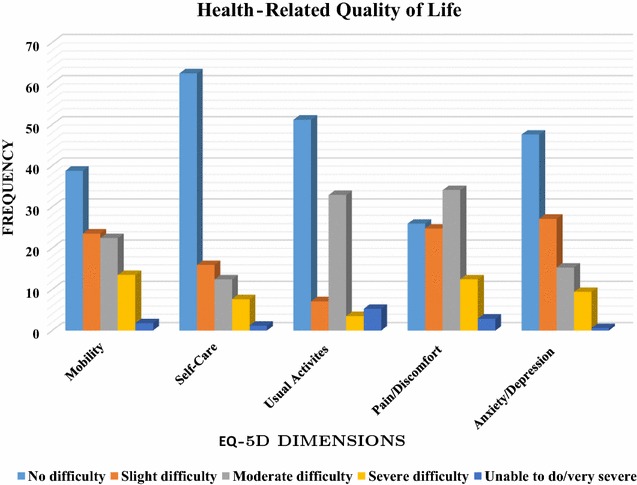
Distribution of health-related quality of life measures in different European Quality of Life scale 5 (EQ-5D) dimensions

The reported HRQOL of this study was measured by using the median EQ-5D-5L index score, which was 0.67 (interquartile range: 0.51–0.84). Cronbach’s alpha for the EQ-5D-5L scale was 0.824, indicating satisfactory internal consistency. The distribution of participants with answers of no problem across the dimensions of EQ-5D was as follows: mobility 66 (38.8%), self-care 107 (62.5%), usual activities 87 (51.2%), pain/discomfort 44 (25.9), and anxiety/depression 81 (47.6%); (Fig. 1). We found that 25 (14.7%) women reported no problems with any dimension of EQ-5D. Furthermore, the median EQ-VAS was 70.00 (interquartile range: 60.00–80.00).

### Univariate and multiple linear regression analyses

As shown in both Tables 1 and 2, there were significant differences between breast cancer patients in relation to patient age, marital status, income level, the current condition of cancer and post-treatment pain (p-value < 0.05). The study also showed no significant differences between breast cancer patients in relation to their educational level, residency, occupation, BMI, and histopathological breast cancer type (p-value > 0.05).

There was a significant moderate negative correlation between pain severity score and EQ-5D-5L index score (r = − 0.58, p-value < 0.001). Also, there was significant moderate negative correlation between pain interference score and EQ-5D-5L index score (r = − 0.604, p-value < 0.001). This study showed a moderate negative correlation between EQ-VAS score on the one hand with pain severity score (r = − 0.46, p-value < 0.001) and pain interference score (r = − 0.53, p-value < 0.001) on the other. Also, the study showed a moderate positive correlation between EQ-5D-5L index score and EQ-VAS score (r = 0.66, p-value < 0.001).

Regression analysis, using QOL score as a dependent variable and the covariates of age, marital status, employment status, income, current condition of cancer, pain severity score, pain interference score, and post-treatment pain as independent variables revealed that patients with high income (p-value = 0.003), patients with lower pain severity score (p-value < 0.001), and lower pain interference score (p-value = 0.018) were independently associated with high QOL. The factors significantly associated with QOL according to multiple linear regression analyses are summarised in Table 3.

**Table 3:** Patients characteristics associated with quality of life in multiple linear regression

Variables	Unstandardised coefficients (B)	S.E	Standardised coefficients (Beta)	P value
Age	− 0.020	0.017	− 0.066	0.241
Marital status	0.052	0.041	0.077	0.205
Income level	0.064	0.023	0.163	*0.006*
The current condition	− 0.023	0.019	− 0.074	0.222
Pain severity score	− 0.011	0.002	− 0.353	*<* *0.001*
Pain interference score	− 0.007	0.001	− 0.350	*<* *0.001*
Post-treatment pain	− 0.010	0.051	− 0.012	0.842

Italic values indicate significance of *p* value (*p* < 0.05)

## Discussion

This study provided an inclusive measurement of HRQOL between breast cancer patients in Nablus, Palestine. In our study, EQ-5D QOL instrument was applied to measure HRQOL. Overall, the main socio-demographic factors related to breast cancer HRQOL were old age, being a housewife, low income, being single, an active or recurrent tumour and post-treatment pain. According to the literature, EQ-5D was also used to assess HRQOL among breast cancer patients in different countries [33–35]. EQ-5D measured improvements and deteriorations in HRQOL after treatment [36]. Thus, EQ-5D seemed an appropriate tool for evaluation of HRQOL and possible interventions to improve QOL among breast cancer patients, especially after treatment [36].

In our study, the EQ-5D score median among breast cancer survivors in Palestine was 0.67 (interquartile range: 0.51–0.84); this compared to other studies which used the same instrument in Iran, Holland and Sweden, with the following results: 0.69 ± 0.22 [33], 0.72 ± 0.29 at the end of treatment, 0.57 ± 0.29 12 months after the end of treatment [34], and 0.70 (95% confidence interval (CI): 0.63–0.75) [35], respectively. Several socioeconomic factors and factors related to the healthcare system could affect HRQOL in many aspects. Some of these variations resulted from differences in socio-demographic and clinical characteristics of the participants such as: age, residency, marital status, occupation, income level, current condition of the tumour, and post-treatment pain.

According to our results, increased age was associated with lower HRQOL among breast cancer patients. Similarly, many previous studies concluded the same findings [37]. One of these studies, a study from Malaysia, stated that QOL in breast cancer strongly varies by age as an important component of general health status [38]. Younger patients reported significantly better HRQOL compared to older ones, possibly due to the short duration of disease and fewer complications.

One possible explanation is that older patients age, as the disease progresses, and will experience a poor social life with an increased rate of depression and physical inactivity, which could lead to high pain and fatigue level and thus lower QOL scores [39]. This observation was also reported by Merom et al. [40], who described physical inactivity as being high among Palestinian women. Thus, older patients who present with more symptoms of depression and anxiety and physical inactivity will contribute to the lower QOL [41].

Our data showed in relation to marital and financial status that being single and having a low income level were significantly associated with poor QOL (i.e. lower EQ-5D scores). Low income level was also confirmed as being an important factor related to impaired HRQOL among breast cancer patients in other studies [37]. Another study about HRQOL in breast cancer patients was performed in Lithuania, and showed a good QOL level in patients who were married and lived in families with fewer financial difficulties compared with patients who were single and with poor economic status [42]. Also, being single was negatively associated with HRQOL, these results further support the idea that a strong family relationship, close communication, and positive emotional and social support given by the partner had a significant effect on improving QOL in breast cancer patients [42–44]. Therefore, good social support from family and friends and good financial status may significantly improve the QOL in breast cancer patients [45].

According to our study, current breast cancer condition was significantly related to impaired HRQOL in breast cancer patients. Patients experiencing recurrent cancer or undergoing active cancer treatments reported a lower HRQOL than those who are cancer-free. These findings could be explained by the suggestion that receiving breast cancer treatment will induce post-treatment pain which interferes with patients’ functioning and quality of life [15, 46, 47].

Post-treatment pain in breast cancer patients remains clinically especially during the first few years following treatment [48, 49]. Many patients experience severe post-treatment pain that significantly interferes with their functionality and quality of life [50–52]. All treatment modalities of cancer have the ability to cause pain [15]. The pain aetiology for breast cancer is categorised as: tumour-induced pain, treatment-induced pain such as side effects from chemotherapy, or post-procedural and post-surgical pain, and comorbidity-related pain such as constipation and thrombophlebitis [15, 53, 54]. Among breast cancer patients, the prevalence of post-treatment pain may be much higher; according to our study, 87.6% of patients with breast cancer suffered from post-treatment pain. These results were much higher than the findings reported by Forsythe and colleagues, with more than 30% of breast cancer patients reporting above average pain after treatment [55].

In this study, we found that post-treatment pain was significantly associated with low levels of HRQOL. Patients who experienced pain after breast cancer treatment reported lower EQ-5D scores. These findings were confirmed by the results of Kroenke and Theobald [56]. Many studies also demonstrate that pain in breast cancer is associated with a lower HRQOL, especially in high levels of pain severity and pain interference, while good pain management led to an improved QOL [42, 57, 58]. Pain interference mostly affected normal work, walking ability, mood, sleep and general activity. Our results showed that post-treatment pain did not negatively affect HRQOL alone, but was also the most significant determinant of HRQOL among breast cancer patients. In summary, our results indicated that patients who had chronic pain after breast cancer treatment reported fatigue, anxiety, depression, sleep disturbances and impaired HRQOL. Receiving a combined treatment for breast cancer, such as surgery, chemotherapy, and regional radiotherapy, was related to a higher risk of developing chronic pain. The identification of those breast cancer patients who are at high risk of developing chronic pain after the completion of breast cancer treatment is hugely important in order to provide adequate pain relief, establish interventions which aim to reduce the adverse consequences of breast cancer treatment, restore functionality and support healthy life in long-term breast cancer patients.

### Strengths and limitations

This study has many strong points in that it is the first study about HRQOL among breast cancer patients conducted at West Bank in Palestine; also, the information was gathered by face-to-face interviews to get more reliable and complete data. Also, the current study measured the impact of pain on HRQOL by using the global BPI and EQ-5D scales. However, in our study, we found a number of limitations that should be focused on. One of these limitations, the cross-sectional study type of this study, may prevent us from developing a good cause—effect relationship between post-treatment pain and HRQOL. Another limitation is that this study was held in Nablus city, which represents only one section of the entire Palestinian West Bank. Lastly, gathering study data via a face to-face interviews may have a negative outcome as the researchers can influence participant’s answers, leading to less reliable data.

## Conclusions

This is the first study to present important data regarding QOL by using the EQ-5D-5L instruments that may help healthcare providers to identify patients with breast cancer who are at risk of low QOL. Our current study identified a number of significant associated factors that should be considered when dealing with breast cancer patients. Breast cancer patients with high pain severity, higher degrees of pain interference, and low income levels all reported with poor HRQOL. Our findings are likely to be important for educators, doctors, and clinics dealing with breast cancer patients. Healthcare providers and policy makers have to be alerted to the low QOL in patients with a low income level, and in patients with post-treatment pain, especially those in a state of severe pain and state of pain interfering with daily life, in order to improve their HRQOL.

## Abbreviations

HRQOL: health-related quality of life
EQ-5D-5L: five-level EuroQol five-dimensional instrument
BPI: Brief Pain Inventory
IRB: Institutional Review Board
SD: standard deviation
